# Risk of HIV Infection and Lethality Are Decreased in CCR5del32 Heterozygotes: Focus Nosocomial Infection Study and Meta-analysis

**Published:** 2012

**Authors:** S.A. Borinskaya, Zh.M. Kozhekbaeva, A.V. Zalesov, E.V. Olseeva, A.R. Maksimov, S.I. Kutsev, M.M. Garaev, A.V. Rubanovich, N.K. Yankovsky

**Affiliations:** Vavilov Institute of General Genetics, Russian Academy of Sciences; Moscow Institute of Physics and Technology; Ministry of Health and Social Development of the Republic of Kalmykia; Blood Centre of the Republic of Kalmykia; Rostov State Medical University; Ivanovsky Research Institute of Virology, Russian Academy of Medical Sciences; Faculty of Biology, Lomonosov Moscow State University,

**Keywords:** HIV, nosocomial infection, lethality risk, infection risk, chemokine receptor gene, allele*CCR5del32*, meta-analysis

## Abstract

*CCR5del32 *Homozygous deletion in the chemokine receptor R5 gene provides almost complete protection to individuals against HIV infection. However, data relating to the protective effect for*CCR5del32 *heterozygous individuals have been contradictory. The frequency of the*CCR5del32*allele in population control cohorts was compared with that of a group of children (27 Kalmyks and 50 Russians) infected by G-subtype HIV-1 in a nosocomial outbreak. The frequency of the*CCR5del32*allele was shown to be lower among the infected children in comparison with that of the control group; however, the difference was small and statistically insignificant. Similar results were obtained in a number of earlier studies. The insignificance of the small differences could be a result of one of two reasons. (i) The fact that there is no protective effect of the heterozygous state, and that the phenomenon depends only on the fluctuation of allele frequencies. In this case, there would be no differences even if the infected cohort is enlarged. (ii)The protective effect of the heterozygous state is real; however, the size of the studied cohort is insufficient to demonstrate it. In order to discern between these two reasons, a meta-analysis of data from 25 published articles (a total of 5,963 HIV-infected individuals and 5,048 individuals in the control group, including the authors’ own data) was undertaken. A conclusion was drawn from the meta-analysis that the*CCR5del32 *allele protects individuals against the HIV infection even in a heterozygous state (*OR=*1.22, 95%*CI*=1.10–1.36). The risk of HIV infection for*CCR5 wt/del32 *heterozygotes was lower by at least 13% as compared to that for wild type*CCR5 wt/wt*homozygotes*. *Prior to this study, no data of the type or any conclusions had been published for Caucasians. The mortality rate in the 15 years following the infection was found to be approximately 40% lower for*CCR5del32 *heterozygotes in comparison with that for the wild type homozygotes in the studied group. The size of the studied group was insufficient to claim difference validity (*OR*=2.0;*p*= 0.705), even though the effect quantitatively matched the published data. The features of the meta-analysis influencing the threshold level and the statistical validity of the effects are being discussed. The level of the*CCR5del32 *protective effect on the chances to be infected with HIV and on the outcome of the HIV infection was assessed for various ethnic groups.

## INTRODUCTION

**Fig. 1:**
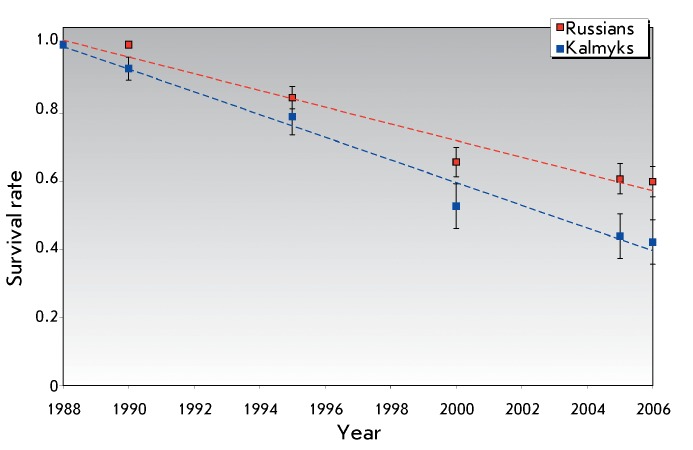
Dynamics of the survival rate for individuals with nosocomial HIV infection: Russians (Rostov-on-Don – 107, Elista – 13 individuals) and Kalmyks (Elista – 57 individuals).

Having started with a single case detected in 1981, the AIDS epidemic is now one of the most important health care issues both in Russia and the rest of the world [1]. The evolution of the epidemic in Russia was characterized by the formation of clusters of nosocomial infection that took place in 1988–1989. The outbreak of the infection began with a HIV-infected child at a children hospital in Elista. Antiepidemic measures were not taken, which resulted in the spread of the epidemic throughout medical institutions in Kalmykia, the Rostov and Volgograd districts, as well as Stavropol Territory. A single focus (focus of infection) was responsible for infecting more than 260 children and their mothers [2, 3]. Many of them have long passed away ( *Fig. 1* ).

Some HIV-infected patients demonstrated rapid progression of the disease (2–3 years) and emergence of AIDS symptoms, whereas in other patients HIV symptoms took a considerable length of time to manifest. The differences in the rate of disease progression may be the result of a combination of external factors (infection conditions, concomitant diseases, ongoing treatment) and the individual genetic characteristics of a patient [4].

Among the human genes affecting the progression of the HIV infection, the *CCR5* gene that encodes the CC chemokine receptor 5, which mediates HIV binding to the cell membrane and penetration of certain viral strains into the cell, plays the most significant role [4]. A 32 bp deletion in the *CCR5* gene ( *CCR5del32* (rs333)) results in the production of a nonfunctional protein. Individuals with homozygous deletion bear no functional CCR5 receptors, whereas the density of these receptors in individuals with heterozygous deletion is reduced. The CCR5del32 allele occurs predominantly in European populations. Its frequency is the highest in northern European countries (up to 15–18%). It is lower than 3–5% in most Asian populations. This allele is absent in almost all individuals of the native populations of America and Oceania [5–7].

CCR5del32 homozygous individuals (their proportion in European populations is equal to 1–2%) show high, although not absolute, resistance to infection. There are very few CCR5del32 homozygous individuals among HIV-infected individuals. Only 12 cases have been reported among the more than 20, 000 patients examined; for most of them, the virus was tropic for CXCR4, but not CCR5 [8–13]. The protective effect of *CCR5del32* homozygosity has been confirmed both in a number of epidemiological studies (an increased homozygote frequency among HIV-negative individuals who had a risk of infection) and via *in vitro * infecting of CD ^+^ cells derived from individuals of various genotypes [14].

The protective effect of *CCR5del32* heterozygosity also manifested itself in the development of AIDS symptoms in HIV-infected individuals. It is not unlikely that the possibility of a symptomatic undiagnosed bearing of HIV by CCR5del32/+ heterozygotes may facilitate the spread of the infection. The viral load in HIV-infected *CCR5del32* heterozygous individuals is lower, the CD4+ T cell count decreases at a slower rate, and AIDS symptoms develop slower both in adults [8, 11, 13–17] and in children (most of whom were perinatally infected) [18]. The frequency of *CCR5del32 * heterozygosity was considerably higher in the group of individuals who were infected in the 1980s and survived a period of 10 years post-infection [11].

However, data indicating that *CCR5del32 * heterozygosity protects against HIV infection remain controversial. In a number of studies, the heterozygosity frequency among infected individuals has been found to be higher than that among the healthy ones who were at risk of infection, or than that of the total sample of the same population; points which may indicate the fact that individuals with the *CCR5wt/del32 * genotype have partial HIV-1 resistance [10, 12]. This has not been observed in other studies; the difference between the frequencies of *CCR5wt/del32 * heterozygotes and/or *del32 * alleles between groups of HIV-positive and HIV-negative individuals have been either absent or statistically insignificant [8, 19–21]. In this study, the effect of *CCR5del32* heterozygosity on the survival rate of children with focus nosocomial HIV infection and the risk of infection upon transmission route through injection were analyzed. In addition, a meta-analysis of the published data was performed in order to assess the potential decrease in the risk of infection in heterozygous individuals for the *CCR5del32* allele.

## MATERIALS AND METHODS

Blood samples from the collection of the Biotechnology Laboratory (Ivanovsky Institute of Virology, Russian Academy of Medical Sciences) were used in this study. The samples were obtained as a result of planned medical examinations of individuals with nosocomial HIV infection, during the period spanning 1991–2007. Consent letters were obtained from the parents of each of the examined children giving permission to use some of the samples obtained for research purposes. This sample of HIV-infected patients is unique, since there is no variability of infection development associated with differences in viral strains. All of the patients were infected with the same viral strain (HIV-1 subtype G) originating from the original child that had been infected (focus nosocomial infection) [22, 23]. Furthermore, most patients belonged to two ethnic groups (Russians and Kalmyks), thereby reducing the possible influence of genetic heterogeneity in each cohort. Anonymous data on patients’ birth dates and death dates in cases of fatal outcomes were obtained for 107 HIV-infected patients in the Rostov district (all Russians) and 60 HIV-infected patients from Elista (47 Kalmyks and 13 Russians). Blood samples of HIV-infected children (50 Russians and 27 Kalmyks, age varied from less than 1 year to 16 years; median age 2.7 years) were used for the study. Blood samples taken from healthy volunteers were used as control samples. The first control group consisted of students of the Rostov State Medical University (the majority of whom were born in 1986–1990). According to the results of the survey, they were second-generation Russians and were born in the Rostov district. The second control group consisted of Kalmyks living in Elista (ethnicity was established based on the survey results). Blood samples were collected in full compliance with the informed-consent procedure. The genetic study project obtained approval from the Ethics Committee of the Institute of the Institute of General Genetics, Russian Academy of Sciences.

Genomic DNA was extracted from venous blood samples (up to 50 µl) using a commercial kit DNAPrep (IsoGene, Russia) at the Biotechnology Laboratory of the Institute of Virology, Russian Academy of Medical Sciences (equipped for handling infected samples), according to the manufacturer’s procedure.

Genotyping was performed using PCR amplification of DNA samples. The primers and amplification conditions were described in [24]. PCR products underwent 2% agarose gel electrophoresis in order to determine the length of DNA fragments. 

**Estimated value of the protective effect of CCR5del32 allele in the heterozygous state**

The effects observed for all samples were uniformly characterized using the odds ratio measure ( *OR* ), which was calculated as the ratio between the chance of bearing the *wt/wt * genotype in HIV-positive and HIV-negative individuals: 
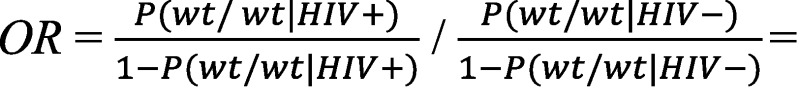
 
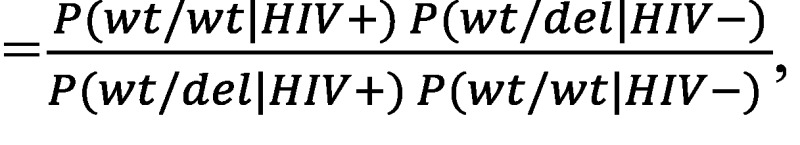


where *P* (*| HIV+) and *P* (*| HIV–) are the genotype frequencies in the samples of infected and healthy individuals, respectively. The risk ratio ( *RR* ), which is determined as the ratio between morbidities for various genotypes, was assessed using the following formula: 
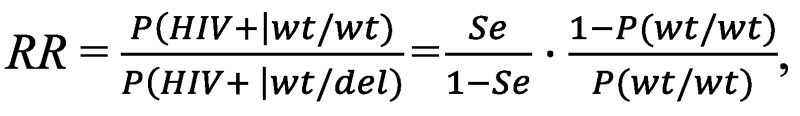


where *Se* is the test sensitivity for disposition, i.e., the frequency of the risk *wt* / *wt* genotype in patients, and *P* ( *wt * / *wt* ) is the population frequency of the risk genotype.

The statistical significance of frequency differences was assessed using the two-tailed Fisher’s exact test.

The meta-analysis was conducted using a freeware package of statistical programs for epidemiologists WinPepi v.10 (2010) [25]. The package allows one to estimate the median *OR* value based on the fixed effects model (Mantel–Haenszel test) and the random effects model (Der Simonian–Laird method). The choice between the two models was made based on a dataset analysis (Cochran’s Q test).

## RESULTS

**Allele and genotype frequencies in HIV-infected patients and in the control groups**

*CCR5 *genotyping was carried out for each child from the sample of children with nosocomial HIV infection and the control group individuals; *CCR5del32 * allele bearers were revealed ( *Table 1* ). Genotype distribution in all groups did not differ significantly from the Hardy–Weinberg equilibrium. The *CCR5del32* allele frequency was first ascertained in the Kalmyk population and was equal to 0.021 ± 0.012. The low allele frequency in the Kalmyk population correlates with its frequency in the neighboring population of the Caucasus (3–5%) and the low frequency in the populations of Central Asia of close origin with the Kalmyks (e.g., 1.1% for the Mongolians in China [26]). No bearers of this allele were detected in the sample consisting of 27 HIV-infected Kalmyk children (the differences from the frequency in the control group were insignificant: *p*  = 0.558, based on the exact Fischer’s test).

**Table 1:** Distribution of the genotype and allele frequencies over the CCR5 gene in HIV-infected children and in the control samples

Group	N	Number of individuals (genotype frequencies, %)			Allele frequency and statistical error (±SE)		Comparison of the HIV+ and control groups
		wt/wt	wt/del	del/del	wt	del	
HIV, Kalmyk children	27	27	0	0	1	0	OR = 2.85p = 0.558
Control, Kalmyks in Elista	70	67 (95.71)	3 (4.28)	0	0.979 ± 0.012	0.021 ± 0.012	
HIV, Russian children	50	39 (78.0)	11 (22.0)	0	0.890 ± 0.031	0.110 ± 0.031	OR = 1.21p = 0.690
Control, Russians in the Rostov district	99	73 (73.7)	25 (25.3)	1 (1.0)	0.864 ± 0.024	0.136 ± 0.024	

**Table 2:** The frequencies of the CCR5del32 (rs333) allele in groups of Russians and Kalmyks

Population	N	CCR5del32 allele frequency	CI_95%_	Reference
Russians: Leningrad district	33	0.166	0.083–0.300	[27]
Kostroma	54	0.157	0.091–0.252	[28]
St. Petersburg	50	0.130	0.069–0.223	[29]
Moscow	83	0.139	0.088–0.208	[30]
Moscow	176	0.122	0.088–0.164	[31]
Ryazan	78	0.12	0.072–0.188	[32]
Lipetsk	48	0.104	0.045–0.192	M.M. Garaev’s own data
Novosibirsk	53	0.104	0.051–0.187	[33]
Lys’va	186	0.100	0.070–0.138	[34]
Moscow, ethnicity not specified	171	0.091	0.062–0.129	[35]
Rostov-on-Don	99	0.136	0.089–0.198	This study
Russian children, HIV	50	0.110	0.054–0.198	This study
Kalmyks	70	0.021	0.004–0.063	This study
Kalmyk children, HIV	27	0	0–0.073	This study

According to the published data, the *CCR5del32 * allele frequency in Russians varies from 0.104 to 0.157 ( *Table 2* ) (see review in [7]).The control group was formed of volunteers (the students of the Rostov State Medical University) since most infected Russian children within the sample under study were undergoing treatment in Rostov-on-Don hospitals; whereas the *CCR5del32 * allele frequency in Russians of the Rostov district was unknown. According to the survey results, they were second-generation Russians and had been born in the Rostov district. The *CCR5del32 * allele frequency in this group was equal to 0.136 = 0.024, which lies within the frequency variability range in various geographic groups of Russians. The *CCR5del32* allele frequency in HIV-infected Russian children turned out to be slightly lower (0.110 ± 0.031); however, the differences were of no significant degree ( *OR*  = 1.21, *p*  = 0.69).

For such a small sample size, the lower *CCR5del32 * allele frequency in HIV-infected individuals as compared with that in the control group may be a random effect or result from the protective action of this allele.

**Fig. 2:**
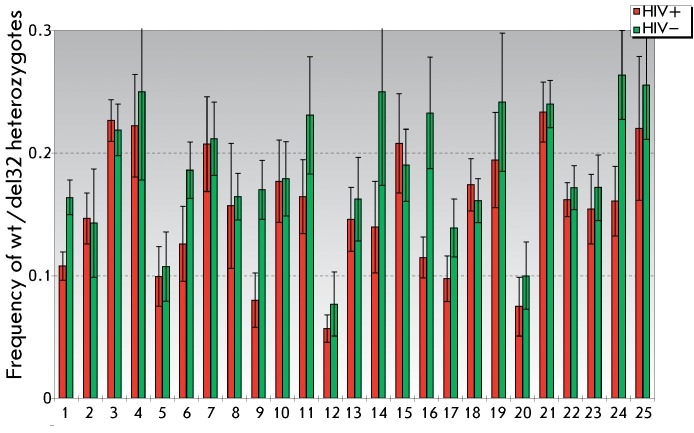
Frequencies of heterozygous bearers of the CCR5del32 allele among HIV-infected (HIV+) and healthy (HIV–) individuals.1 – Belgians and French [10]; 2 – Swiss (HIV-infected [36], control group [37]); 3 – Euro-Americans [38]; 4 – Danes [39]; 5 – Italians, Milan [40]; 6 – Australians [41]; 7 – Finns [42]; 8 – Slovenians [43]; 9 – Spaniards, Asturias [44]; 10 – Moscow residents [19]; 11 – Russians (HIV-infected – Muscovites, control – Russians, Ryazan) [20]; 12 – Spaniards, southern Spain [45]; 13 – Spaniards [46]; 14 – Hungarians [47]; 15 – Russians, Perm district [34]; 16 – Euro-American females [48]; 17 – Germans, Munich [49]; 18 – Euro-Americans, Seattle [50]; 19 – Poles [51]; 20 – Italians [52]; 21 – Estonians [21]; 22 – Germans [13]; 23 – Slovaks [53]; 24 – Poles, Szczecin [54]; 25 – Russians, Rostov district (the present study).

In a number of studies, data on the lower *CCR5del32 * allele frequency and/or lower *wt/del32 * zygote frequency among HIV-infected individuals as compared with the population control group have been obtained. In many cases, these differences do not reach any significant level, whereas an opposite ratio between the frequencies has been revealed in some studies ( *Fig. 2* and the database of allele frequencies (Genome Analysis Laboratory, Institute of General Genetics, Russian Academy of Sciences), http://vigg.ru/institute/podrazdelenija/otdel-genomiki-i-genetiki-cheloveka/laboratorijaanaliza-genoma/allefdb/ccr5-hiv/). The meta-analysis of the published data was performed in the groups of HIV-infected patients and control groups in order to assess the eventuality of a protective effect of heterozygosity for *CCR5del32* .

**Meta-analysis: does heterozygosity for **

**Table 3:** Mortality rate (by 2006) in the samples studied depending on genotype

Genotype	Russians	Kalmyks
wt/wt	12 of 39 (30.8%)	11 of 27 (40.7%)
wt/del32	2 of 11 (18.2%)	–
Total	14 of 50 (28.0%)	11 of 27 (40.7%)

*CCR5del32* **reduce the risk of infection?**

For the meta-analysis, articles comparing the allele and genotype frequencies in the samples of HIV-infected patients and the corresponding control samples of uninfected individuals were selected from over 360 articles found in the PubMed database upon inquiry “CCR5 AND deletion AND HIV” (September 2011). The publications studying Asian, African, and Latin American populations with a *CCR5del32* allele frequency of 1–3% or lower were eliminated from the analysis.

As a result of the differences in the *CCR5del32 * allele frequencies in populations of European origin (from 5–8% in southern Europe to 15–18% in northern Europe) [7], the ethnicity of the control group individuals (and in some cases, their membership in subgroups within an ethnic group) has to closely match the ethnicity of the group of infected individuals. Therefore, the publications in which the ethnicity of the groups was not indicated or the samples were not ethnically homogeneous were also eliminated. A total of 25 Caucasian groups, including our sample, were selected for the meta-analysis: 5,967 HIV-infected individuals and 5,410 control group individuals ( *Table 3* ).

The frequency of homozygous deletion bearers was 4 of 5,967 HIV-infected individuals against 63 of 5,410 individuals in the control group. This ratio corresponds to *OR*  = 17.6 at *p*  = 4.4 × 10 ^–16^ . In this case, the relative risk value is approximately equal to the *OR* value; i.e., the infection probability of deletion homozygous individuals was lower than that of the bearers of the other genotypes by a factor of 17.6. Close estimations of the protective effect of homozygocity for deletion were obtained in separate studies of Euro-Americans, where the groups of seronegative individuals were compared with groups of seropositive individuals and the population control groups [9, 38], and in some of the other studies [10, 11]. Therefore, *CCR5del32/ CCR5del32 * homozygous individuals were eliminated from the subsequent analysis. The ratio between heterozygous bearers of the *CCR5del32 * allele and the individuals without this allele (i.e., the ratio between the *wt* / *CCR5del32 * and *wt/wt * genotypes in the groups of HIV-infected individuals and the population control group) were considered to assess the risk of infection.

It was demonstrated via a comparison of the genotype frequencies that the frequency of *wt* / *CCR5del32 * heterozygotes in HIV-infected individuals was higher than that in healthy individuals in only 4 out of 25 studies ( *Fig. 2* ). Based on the assumption that the effect was random, the probability of the event “the heterozygote frequency in affected individuals is higher than that in healthy ones in no more than 4 studies out of 25” was equal to 4.7 × 10 ^–7^ (similar to the probability of getting no more than four heads after a coin is tossed 25 times).

**Fig. 3:**
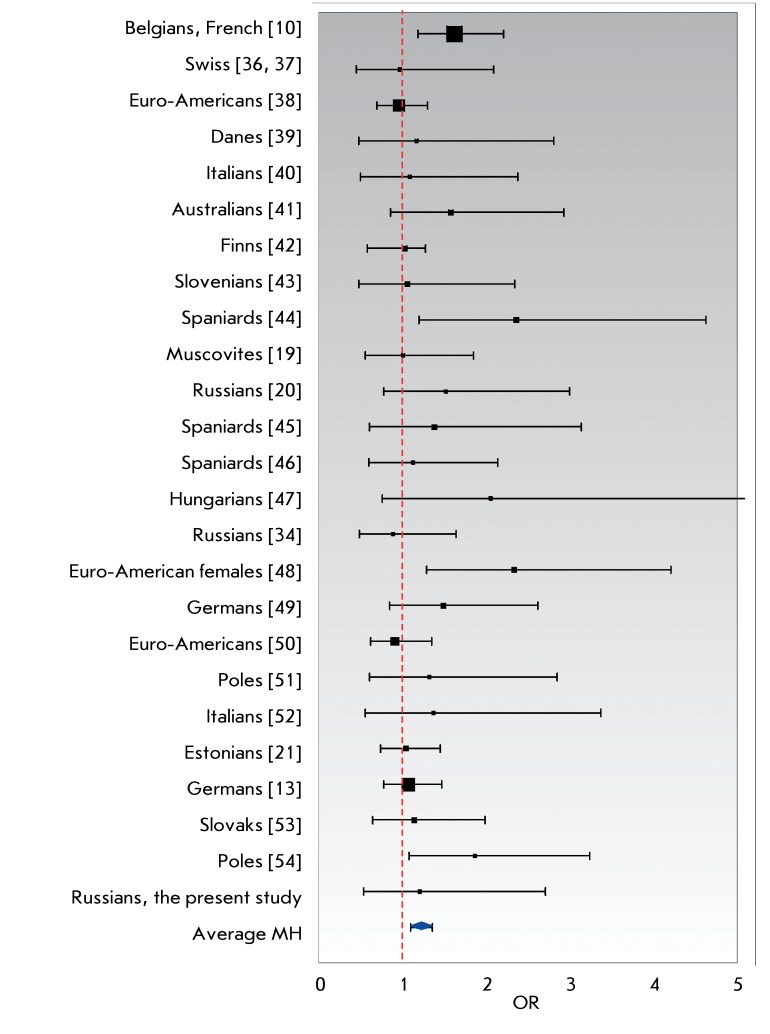
The estimated odds ratios (OR) and the corresponding 95% confidence intervals for 25 samples of Caucasians (specified in the legend to Fig. 2). The vertical dashed line corresponds to OR = 1 (no effects). The points on the right-hand side of this line indicate the protective effect of the wt/CCR5del32 genotype. The size of the square markers is conventionally proportional to sample size. The lower diamond-shaped marker corresponds to the Mantel–Haenszel (MH) estimate of the averaged OR.

The odds ratio ( *OR* ) was determined for each sample; the *OR* values were then averaged with allowance made for the population size and degree of homogeneity of the effects. The results are presented in graphical form ( *Fig. 3* ).

It was found via a meta-analysis carried out on the basis of the results of 25 studies that the *wt* / *CCR5del32 * heterozygote frequency against that of *wt/wt* homozygotes in the samples of HIV-infected individuals was reliably lower than that in the control ( *p = * 0.0002 with the two-tailed Fischer’s exact test and *p = * 0.00018 with the χ ^2^ test). With the Cochran’s Q test, heterogeneity of the data was insignificant: χ ^2^ *= * 25.29,( *p * = 0.39 *).* The variability fraction caused by the heterogeneity of *OR* values *I ^2^  = * 5.1% ( *CI*_95%_ *= * 0–36.4%). This value is considerably lower than the critical value (50%), which allows one to use the “fixed effects model,” employing the Mantel–Haenszel test (MH average) for averaging the *OR* values. The total value of the effect is appreciably low: *OR = * 1.22, *CI _95%_  =* 1.10–1.36 *.* However, these estimations have a large stability margin: 28 studies, in which the genotype frequencies would be the same for the samples of HIV-infected individuals and in the control group ( *OR* = 1 *), * need to be added to reduce the total value of the effect to an insignificant level *OR*  = 1.

**Fig. 4:**
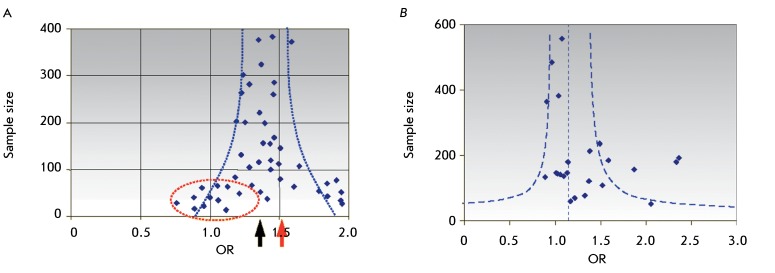
Dependence of the effect intensity (OR) on the composition of the samples selected for the meta-analysis (funnel plot). The strong asymmetry of this plot may indicate publication bias. (A) – The hypothetic distribution of the studies over the size of the samples studied and effect intensity. For the elimination of less willingly published results obtained using small samples with weak effects (dots in a dashed oval), the OR value turns out to be overestimated (red arrow) with respect to the real OR (black arrow). (B) – Distribution of the studies over sample size and effect intensity for 25 publications selected for the meta-analysis in this study (insignificant asymmetry).

The possibility of publication bias is usually taken into account when performing a meta-analysis. The publication bias is a result of the fact that the researchers and journal editorial boards are more willing to publish positive rather than negative or “zero” results. Moreover, studies with significant effects obtained using small samples are often published. All of these facts may result in the overestimation of the averaged effect value in a meta-analysis ( *Fig. 4A* ). Constructing the dependence of the effect value ( *OR* ) on sample size (a funnel plot) is the standard method for checking for data symmetry. A strongly asymmetric plot may attest to selective presentation of the data in publications. In our case, asymmetry is insignificant ( *Fig. 4B):* the Kendall rank correlation between *OR* and the sample size is equal to 0.21 at *p*  = 0.187; asymmetry based on the regression test [55] is unreliable ( *p*  = 0.148).

Thus, a statistically significant, although weak, protective effect of heterozygocity for the *CCR5del32 * allele with respect to HIV-infected individuals (OR = 1.22 at p = 2 × 10 ^–4^ ) was established by the meta-analysis, which included our own experimental data. An appreciably low *OR* value explains the reason why no significant differences in heterozygote frequencies between the HIV-infected individuals and those from the control groups have been found in the majority of articles. The data demonstrate that at a deletion allele frequency of 10% and *OR*  = 1.22, the significant effect ( *p*  = 1.22 at 80% strength) can be detected only when the total sample size is 4,500 (2,250 affected and 2,250 healthy individuals).

The estimated value *OR*  = 1.22 does not mean that the risk of infection in the bearers of the *wt/wt* homozygous genotype is higher than that in the bearers of the *CCR5del32 * allele by 22%. *OR* is the ratio between the chances, but not the risks of infection. The risk ratio parameter ( *RR* ), which is determined as the ratio between morbidities for various genotypes, cannot be directly estimated in context-dependent case-control studies. One may only propose various indirect estimations of RR based on *OR* and population allele frequency values or morbidity data [56]. Moreover, the inequality *OR*  ≥  *RR * is always valid. According to the results of this meta-analysis, the corresponding estimations are as follows: *SE*  = 0.851 and *P* ( *wt/wt* ) = 0.835; therefore, RR = 0.13. Thus, the infection probability for *wt/wt * homozygous individuals is higher than that for the bearers of the *CCR5del32 * allele by at least 13%.

Our estimation is based on a comparison of the ratios between the *wt/CCR5del32 * and *wt/wt * genotype frequencies in HIV-infected individuals and in the population control group. It is evident that the HIV-infected individuals had contacted the virus and had been infected; whereas the population control individuals had not contacted the virus (the fraction of the individuals who contacted the virus and/or HIV-infected ones is assumed to be negligibly small in the populations of European origin under study). The protective effect of heterozygosity can be assessed more precisely by using the control group consisting of individuals who had contacted the virus but remained HIV-negative. However, the fraction of individuals in the existing high-risk groups (haemophiliacs; sexual partners of HIV-infected individuals; injection drug users; men practicing receptive penile–anal contacts with men (MSM); prostitutes) who had contacted the virus differs largely and sometimes cannot be determined. The random-effects model can be used when carrying out a meta-analysis of the results of these studies. However, because of the sample heterogeneity, *OR* estimations to a significant extent show the probability of contacting the virus in various risk groups rather than showing the direct protective effect of the allele in the group of individuals who contacted the virus.

This phenomenon can be illustrated by comparing the frequencies of homozygous genotypes for the deletion allele among uninfected individuals in two risk groups: haemophiliacs [57] and MSM individuals [38]. Among the uninfected individuals in the MSM group, the *CCR5del32* / *del32 * homozygote frequency was equal to 4.5% (5 of 11 individuals); whereas the frequency among the haemophiliacs was equal to 16.3% (7 of 43 individuals), given the fact that the population frequency of these homozygotes in the Caucasian populations is less than 1–2%. The differences in the frequencies of individuals with homozygous deletion are statistically significant in the two risk groups ( *p* = 0.038, two-tailed Fischer’s test). A higher *CCR5del32/del32 homozygote * frequency among haemophiliacs is accounted for by a higher risk of infection; for the individuals receiving intense therapy with blood-based products in 1978–1985, it was equal to 94% [57]. Since almost 100% of patients who were administered blood-based samples contacted the virus, it can be assumed that the higher frequency of homozygotes for the deletion allele (i.e., a more pronounced protective effect) cannot be achieved because of the genetic heterogeneity of the indicator of resistance to infection with macrophagotropic HIV strains [58], similar to how the selection of protective alleles of different genes ensuring resistance to infection (e.g., malaria) is carried out in the same population under natural conditions.

**Effect of heterozygosity for the CCR5del32 allele on the survival rate of HIV-infected individuals**

The variability in the progression of the HIV infection into AIDS and the mortality rate were observed previously for both samples (for Russians and Kalmyks). The variability is caused by nongenetic factors (infection age, which varies from several months to 14 years in these samples; the median age is 2.5 years), intensity of parenteral interventions, and concomitant diseases [3].

By 2006, the mortality rate in the sample studied was equal to 32.5% (25 of 77 individuals). The mortality rate was 28.0% (14 of 50) among the infected Russian children and 40.7% (11 of 27), among Kalmyk children. By 2006, the rate of mortality in Russians in the studied group was lower than that for the Kalmyks by 31.2%; however, these differences were insignificant for the given sample sizes ( *p*  = 0.311). Nevertheless, the regressions describing the general mortality dynamics differ to a significant degree: the slope angle of the regression for Russian children is equal to –0.016 ± 0.004, against –0.025 ± 0.002 for Kalmyk children ( *p*  = 0.02, two-tailed Z test).

The effect of the carriage of the *CCR5del32 * allele on the survival rate of infected individuals was tested, as well as whether the differences in longevity post-infection could be linked to the differences in the *CCR5del32* frequency in the two ethnic groups.

In the sample subjected to study, no deletion allele was detected in HIV-infected Kalmyk children; all of them had the *wt/wt* genotype. Among the infected Russian children with the *wt/wt* genotype, the rate of mortality was 30.8% (12 of 39%), and 18.2% (2 of 11) among the bearers of the CCR5del32 allele ( *Table 3* ). Thus, the mortality rate in Russian children with the *wt/CCR5del32* genotype 15 years after the infection was lower by 40.9% compared with that of the children without the deletion allele, although these differences are statistically insignificant ( *OR*  = 2.0; *p* = 0.705). The limited sample size makes it impossible to accept or refute the hypothesis that the differences in longevity in the two ethnic groups are caused by the differences in the *CCR5del32* allele frequencies. Nevertheless, it should be noted that our insignificant estimations literally line up with previously published data. According to the results of the meta-analysis of 19 cohorts of HIV-infected individuals (a total of 1,635 Caucasians), the protective effect of heterozygous bearing of the *CCR5del32* allele manifested itself in a 39% decrease in the risk of death [17]. In order to attain statistical significance at this level of the effect ( *OR*  = 2), the sample size for the infected individuals has to be at least 550 individuals at a mortality rate of 30% and 400 people at the instant when mortality is as high as 60%.

Thus, in the study that focused on 507 HIV-infected Poles who were observed over a period of 15 years prior to the introduction of antiretroviral therapy, the differences in the rate of mortality between the bearers of the *wt/del32* and *wt/wt* genotypes were equal to 49% (the overall mortality rate being 19%). These differences were statistically significant ( *p*  = 0.026), whereas the differences were insignificant ( *p*  = 0.23) for individuals receiving treatment (442 individuals) [59].

## DISCUSSION

The effect of the heterozygous bearing of the *CCR5del32* deletion allele on the risk of HIV infection in populations of European origin (without allowance for the route of infection transmission, viral serotype, and differences in antiretroviral therapy) was first assessed based on the meta-analysis of published data. The protective effect observed was rather small ( *OR*  = 1.22) but was statistically significant and corresponded to a decrease of at least 13% in the risk of infection in the individuals with the *CCR5wt/del32* genotype, according to the calculations. The small *OR* value explains why the differences between the frequencies of genotypes and/or CCR5del32 allele between the groups of HIV-infected individuals and the control group detected in most articles are statistically insignificant.

The size of the samples that are to be studied in order to demonstrate the reliability of this phenomenon reversely depends on the allelic abundance in the population. In particular, in Chinese populations, where the abundance of the *CCR5del32* allele is lower than that in Europeans, no significant protective effect of heterozygous bearing of *wt/del32* has been detected via a meta-analysis: *OR*  = 1.156 ( *CI _95%_  * = 0.808–1.654) [60].

The *CCR5del32* allele is predominantly present in populations of European origin; its abundance in southern European population groups (Spaniards, Italians, and Greeks) is equal to 5–8%, being as high as 15–18% in northern European population groups (Finns, Estonians, Mordvinians, Tatars, etc.) [7]. In Russians, the frequency of the *CCR5del32* allele is appreciably high (10–17% in different regions), whereas the frequency of this allele is equal to 2% in the other group that was studied (Kalmyks). Can the differences in the frequency of the protective *CCR5del32* allele play a substantial role in the prevention of the HIV infection at the population level or can they be accounted for by the differences in the mortality rate of HIV-infected individuals?

Hypothetically, the population effects caused by the presence of the deletion allele can be assessed as follows. Let us assume *q * to be the frequency of the deletion allele, and *S _ww_* , *S _wd_* , and *S _dd_* – the survival rates of the infected individuals with *wt/wt* , *wt/CCR5del32* , or *CCR5del32* / *CCR5del32* genotypes, respectively. Then, the population average survival rate *S _pop _* is higher than the survival rate of the individuals with the *wt/wt* genotype by the following figure:

ΔS = S _pop_  – S _ww_  = (1–q) ^2^ S _ww_  + + 2q(1–q)S _wd_ + q ^2^ S _dd_ – S _ww_ ≈ 2(S _wd_ – S _ww_ )q.

The terms of *q ^2^* order were neglected in the latter equality. Thus, the protective effect of heterozygous bearing of the *CCR5del32* allele (a 40% decrease in the mortality rate for the infected individuals) for a 10% frequency of this allele provides an 8% decrease in the general death rate for the HIV-infected individuals against the group without bearers of this allele. A decrease in the risk of infection due to the presence of the *CCR5del32* allele in the population is calculated identically. If the probability of infection of heterozygous individuals is reduced by 13%, the general infection frequency for the population is reduced by 3.3%. At a 15% allele frequency, the decrease in the infection rate would be 5.6%; whereas the decrease in the mortality rate of the HIV-infected individuals would be 12%.

To summarize, protection against the HIV infection and the reduction in mortality rates in HIV-infected individuals at the population level is rather small even in groups with a high frequency of the *CCR5del32* allele (15%). In addition to *CCR5del32* , there are other genes which affect both the susceptibility to HIV infection and the course of progression of the HIV infection [61] and have the ability to contribute to interpopulation differences. Thus, Russians and Kalmyks differ in terms of the frequencies of the protective *C/C* genotype at polymorphism in the regulatory site of the interleukine 10 gene * IL* 10–592 A/C *(* 49%in Russians in the Rostov district and 33% in Kalmyks in Elista) and in terms of the frequencies of the protective CCR2-64I allele (12% in Russians of the Rostov district and 23% in Kalmyks in Elista) (the data obtained by the authors have yet to be published). However, the possible contribution of these genes to interpopulation differences in the progression of the HIV infection requires further studies. The authors express the hope that samples with nosocomial infections will no longer be available. 

## Abbreviations

HIV: human immunodeficiency virus
AIDS: acquired immune deficiency syndrome
PCR: polymerase chain reaction
